# Hybrid Feature Selection for Predicting Chemotherapy Response in Locally Advanced Breast Cancer Using Clinical and CT Radiomics Features: Integration of Matrix Rank and Genetic Algorithm

**DOI:** 10.3390/cancers17172738

**Published:** 2025-08-23

**Authors:** Amir Moslemi, Laurentius Oscar Osapoetra, Aryan Safakish, Lakshmanan Sannachi, David Alberico, Gregory J. Czarnota

**Affiliations:** 1 Physical Sciences, Sunnybrook Research Institute, Sunnybrook Health Sciences Centre, Toronto, ON M4N 3M5, Canada; amir.moslemi@ryerson.ca (A.M.); laurentiusoscar.osapoetra@sunnybrook.ca (L.O.O.); asafakis@torontomu.ca (A.S.); lakshmanan.sannachi@sunnybrook.ca (L.S.); david.alberico@sri.utoronto.ca (D.A.); 2 Department of Radiation Oncology, Sunnybrook Health Sciences Centre, Toronto, ON M4N 3M5, Canada; 3 Department of Physics, Toronto Metropolitan University, Toronto, ON M5B 2K3, Canada; 4 Department of Medical Biophysics, University of Toronto, Toronto, ON M5S 1A1, Canada

**Keywords:** LABC, radiomic, hybrid feature selection, CT

## Abstract

Background: Neoadjuvant chemotherapy (NAC) is the important and effective approach to treat locally advanced breast cancer (LABC). The prediction of response to NAC prior to start is an efficient approach to obtaining perspective about the effectiveness of treatment. The objective of this study is to design a machine learning pipeline to predict tumor response to NAC treatment for patients with LABC using the combination of clinical features and radiomics computed tomography (CT) features. Method: A total of 858 clinical and radiomics CT features were determined for 117 patients with LABC to predict the tumor response to NAC treatment. Since the number of features is greater than the number of samples, dimensionality reduction is an indispensable step. To this end, we proposed a novel hybrid feature selection to not only select top features but also optimize the classifier hyperparameters. This hybrid feature selection has two phases. In the first phase, we applied a filter-based strategy feature selection technique using matrix rank theorem to remove all dependent and redundant features. In the second phase, we applied a genetic algorithm which coupled with the SVM classifier. The genetic algorithm determined the optimum number of features and top features. Performance of the proposed technique was assessed by balanced accuracy, accuracy, area under curve (AUC), and F1-score. This is the binary classification task to predict response to NAC. We consider three models for this study including clinical features, radiomics CT features, and a combination of clinical and radiomics CT features. Results: A total of 117 patients with LABC with a mean age of 52 ± 11 were studied in this study. Of these, 82 patients with LABC were the responder group (response to NAC) and 35 were the non-response group to chemotherapy. The best performance was obtained by the combination of clinical and CT radiomics features with Accuracy = 0.88. Conclusion: The results indicate that the combination of clinical features and CT radiomic features is an effective approach to predict response to NAC treatment for patients with LABC.

## Simple Summary

Neoadjuvant chemotherapy (NAC) is an important and effective approach for treating locally advanced breast cancer (LABC). We proposed a machine learning model to predict treatment response prior to initiation, using CT radiomics and clinical features. A robust hybrid feature selection method was introduced to identify the most informative features and optimize the classifier’s hyperparameters. The results demonstrated the effectiveness of combining CT radiomics with clinical features.

## 1. Introduction

Cancer is a major public health concern worldwide and is the second leading cause of mortality in the United States [1]. In Canada, 45% of Canadians are diagnosed with cancer during their lifetime, with lung, breast, prostate, and colorectal cancers being the most common types [2]. Breast cancer is one of the most common cancers [1], and locally advanced breast cancer (LABC) represents a rare subset, accounting for only 5% of all breast cancer cases [3]. LABC is categorized as a heterogeneous disease that includes tumors larger than 5 cm or those affecting the skin or chest wall [4,5]. Additionally, inflammatory breast cancer and cases involving fixed axillary lymph nodes or ipsilateral supraclavicular, infraclavicular, or internal mammary nodal involvement are observed in patients with LABC [4,5]. Statistics indicate lower survival rates for patients with LABC compared to those with early-stage breast cancer [5].

Multimodality treatment, which includes systemic therapy, surgery, and radiotherapy, is the standard approach for LABC [4]. Neoadjuvant chemotherapy (NAC) is a common treatment technique originally used for locally advanced, inoperable tumors to enable surgical resection [6]. NAC aims to achieve downstaging and tumor shrinkage, facilitating the resection of initially inoperable tumors. It can be followed by adjuvant radiotherapy, surgery, endocrine therapy, and targeted therapy [7]. The tumor’s pathological response to NAC has been demonstrated as a critical prognostic factor for both long-term disease-free survival (DFS) and overall survival (OS) in specific patient cohorts [8,9]. Treatment with NAC for patients with LABC leads to a pathological complete response in 15–40% of cases [10].

However, after several months of treatment initiation, the response to NAC is evaluated to assess treatment success. This evaluation is typically based on pathological assessments. The Miller–Payne (MP) grading system is one such metric that measures tumor cellularity by comparing pre-treatment core needle biopsies with post-treatment surgical specimens [9,11]. While the MP grading system and similar techniques are useful for assessing treatment response, they are invasive. Consequently, imaging techniques have gained significant attention as non-invasive methods for evaluating treatment response. Identified imaging biomarkers are employed to predict the tumor’s response to NAC.

Response to NAC treatment for LABC patients is also possible using histopathological analysis and quantitative imaging. Cell proliferation plays a crucial role in distinguishing responders from non-responders, as LABC tumors in responders exhibit less proliferation, which is directly correlated with an increase in apoptosis [12,13]. In this regard, ref. [14] reported a correlation between human epidermal growth factor receptor 2 (HER2) and treatment response to NAC. Furthermore, they showed that the pathological complete response rate of HER2+ tumors is significantly greater than that of HER2-normal tumors [14]. Changes in hemoglobin content are significantly different between responders and non-responders. Studies [15,16] reported that changes in hemoglobin content after one week of therapy differ between complete pathological responders and non-responders. They employed diffuse optical spectroscopy to measure changes in hemoglobin content. Schwarzenbach et al. [17] used DNA and RNA integrity measurements to evaluate tumor response to chemotherapy treatment. In another study, magnetic resonance imaging (MRI) was utilized to measure changes in hemoglobin content to distinguish complete pathological responders from non-responders [18].

As mentioned earlier, quantitative imaging is a method that has demonstrated its capability to distinguish between responding tumors and non-responders. Radiomics features have shown promising results in predicting response to treatment [19]. The term radiomics refers to quantitative features obtained by converting images into mineable data [20]. These radiomics features represent underlying pathophysiological information and can be utilized to train machine learning algorithms with the aim of predicting cancer treatment outcomes [21]. The applications of radiomics features in cancer studies include cancer detection and diagnosis, tracking cancer progression, and predicting treatment response [20].

Although radiomics features provide valuable information for a better interpretation of tumor response, the role of medical imaging and the importance of different imaging modalities are undeniable in outcome prediction and treatment response evaluation. Ha et al. [22] utilized 18F-FDG PET/CT, a fusion of positron emission tomography (PET) and computed tomography (CT), to predict response to NAC in patients with LABC. Hulikal et al. [23] also applied 18F-FDG PET/CT to evaluate tumor response to NAC in patients with LABC, using it directly to assess response without incorporating radiomics features. Antunovic et al. [24] employed radiomics features derived from 18F-FDG PET/CT to discriminate between pathological partial response and pathological complete response to NAC in patients with LABC. Nilsen et al. [25] applied diffusion-weighted MRI (DW-MRI) to assess tumor response to NAC in patients with LABC. Teruel et al. [26] utilized radiomics features derived from dynamic contrast-enhanced MRI (DCE-MRI) to evaluate tumor response to NAC treatment in patients with LABC. Kolios et al. [27] applied radiomics-based MRI textural features combined with machine learning to classify responders and non-responders. The responder group included patients with LABC who exhibited a positive response to NAC. Chen et al. [28] determined radiomics features from contrast-enhanced fat-suppressed T1-weighted images (CE FS T1WI) and employed a machine learning classifier to predict the responder group to NAC in patients with LABC. Osapoetra et al. [29] extracted textural radiomics and texture-derived radiomics features from quantitative ultrasound (QUS) to predict response to NAC treatment. In other studies, QUS texture-derived radiomics features were employed to classify responders and non-responders based on their response to NAC treatment in patients with LABC [30,31].

CT is an imaging modality that provides information about cancerous tumor cells for micro-characteristic analysis by reconstructing 3D volumetric images based on the X-ray attenuation coefficient of organs. Radiomics features derived from CT are considered for tumor cell analysis due to the resolution limitations of CT. The resolution of CT is in the millimeter range, whereas tumor cell size is in the micrometer range. Consequently, radiomics features derived from CT can be employed to train a machine learning classifier to predict response to NAC treatment for patients with LABC.

To this end, Dastjerdi et al. [32] applied CT radiomics features to predict response to NAC in patients with LABC. In another study, they used second-derivative textures of CT to classify patients with LABC into responders and non-responders to NAC [33].

All the studies reviewed above considered only radiomics features to construct a feature space for machine learning. In this study, we hypothesize that an efficient hybrid feature selection with combination of clinical features and radiomics CT features can improve the performance of classifier to predict response to NAC treatment before its initiation in patients with LABC. The objective of this study is to demonstrate that the accuracy of the classifier can be improved in classifying responders and non-responders to NAC treatment using an efficient hybrid feature selection with combination of clinical features and radiomics CT features for patients with LABC.

Although different types of features, including imaging biomarkers and clinical features, provide comprehensive information to train a machine learning model, the curse of dimensionality as a major challenge emerges for the machine learning model, often leading to overfitting. The feature selection approach plays a significant role not only in obtaining the most informative and relative features but also enhancing the performance of the machine learning model in terms of generalizability.

**Figure 1:**
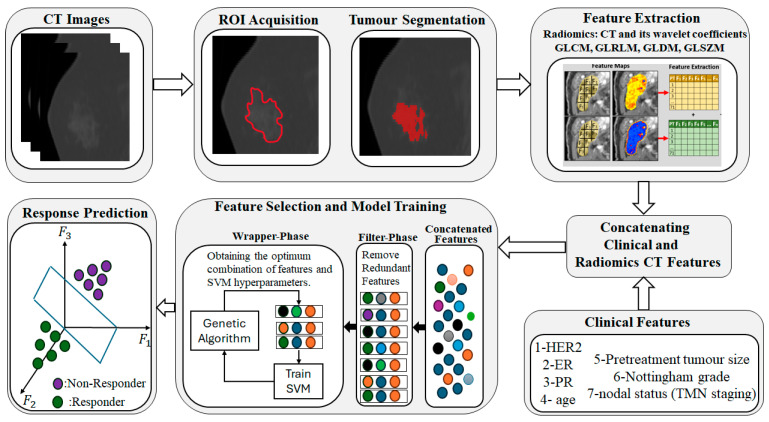
This figure shows each step of this study. CT image acquisition is the first step, and then ROI is determined by an oncologist. In next step, the region of interest (ROI) is segmented and radiomics features are determined. Clinical features and radiomic features are concatenated. A hybrid feature selection is applied to determine the exact top-k features which lead to the best performance of the classifier. In the final step, the support vector machine (SVM) classifier is applied to differentiate the responder from the non-responder patients. Gray-Level Co-occurrence Matrix (GLCM), Gray-Level Run Length Matrix (GLRLM), Gray-Level Dependence Matrix (GLDM), and Gray-Level Size Zone Matrix (GLSZM).

In this study, we propose a novel two-stage feature selection to not only identify the top features but also optimize the hyperparameters of the classifier. In the first stage, we introduce a new filter-based feature selection method based on the matrix rank theorem to remove all redundant features. In the second stage, we integrate a genetic algorithm with an SVM classifier to concurrently acquire the most informative features and the optimal SVM hyperparameters. Figure 1 shows a graphical abstract of this study.

## 2. Materials and Methods

### 2.1. Study Protocol and Data Acquisition

In this study, we followed institutional research ethics guidelines at Sunnybrook Health Sciences Center (SHSC). We included 117 patients with LABC who received NAC treatment. Among the 117 enrolled patients, 82 (70%) were responders, and 35 (30%) were non-responders. Patient enrollment in the study was conditional upon securing written informed consent. Tumor sizes were assessed using MRI scans conducted as a standard component of patient care.

The cancer diagnoses for all 117 enrolled patients were verified through histopathological analysis of pre-treatment core needle biopsy samples. Essential information, including initial cellularity, tumor subtype, and hormone receptor status expressions (estrogen receptor [ER], progesterone receptor [PR], and HER2), was obtained from biopsy samples. The timeline for completing a full course of NAC treatment was 4–6 months for all patients with LABC.

All patients underwent either lumpectomy or mastectomy surgery, along with adjuvant therapies consisting of radiation, maintenance Trastuzumab for HER2-positive tumors, or endocrine therapy for hormone receptor-positive tumors. Institutional practices were followed as the standard for administering adjuvant therapies.

### 2.2. Pathological Evaluation of Tumor Response

Standard histopathologic techniques were employed to evaluate the tumor response to NAC treatment. We then utilized the modified response (MR) grading system to categorize patients with LABC as non-responders (“NR”) and responders (“R”) using the Response Evaluation Criteria in Solid Tumor (RECIST) metric [34] and the residual tumor cellularity metric [7]. RECIST metric is applied to measure and evaluate the percent change in tumor size between pre-treatment and post-treatment. The changes in tumor size reflect treatment response. MR scores of 1, 2, and 3 represent no reduction in tumor size, a reduction in tumor size up to 30%, and a reduction in tumor size between 30–90%, respectively.

Residual tumor cellularity is another technique to assess the treatment response. In this technique, a 5% threshold is considered for tumor cellularity, and responder and non-responder are identified based on this threshold. Less than or equal to 5% (≤5%) of residual cellularity represents a responder, and a non-responder is considered for treatment. In the RECIST metric, an MR score of 3–5 (greater than 30% tumor size reduction) represents a responder and an MR score of 1–2 (less than 30% tumor size reduction) represents a non-responder. An MR score of 3–5 corresponds to less than or equal to 5% of residual cellularity and an MR score of 1–2 corresponds to greater than 5% of residual cellularity. In this study, we considered both RECIST and tumor cellularity criteria to discriminate between responders and non-responders. Consequently, we have a binary classification task in this study since the labels refer to two classes: responder and non-responder.

### 2.3. Radiomics Feature Determination

The regions of interest (ROI) were manually characterized by a specialist so that CT slices delivered whole tumor information. The multi-slice CT scanner (LightSpeed, GE Medical Systems, Chicago, IL, USA) had the scan parameters of tube voltage: 120 kV, X-ray tube current: 10–367 mA, slice thickness: 2.5 mm, pixel spacing: 0.8 × 0.8 mm, and slice size: 512 × 512; pixels were applied in helical mode. Oncologists segmented the tumor in CT images, and radiomics features were determined by Pyradiomics and PyWavelets Python 3.12.7 (Accessed on 1 July 2025) packages [35]. In this study, we determined features from both original CT images as well wavelet coefficients of CT images. Texture-based CT radiomics features were 24 gray level co-occurrence matrix (GLCM) [36], 16 gray level run length matrix (GLRLM) [37], 16 gray level size zone matrix (GLSZM) features [36], 14 gray level dependent matrix (GLDM) features [36], 5 neighboring gray level dependence matrix (NGLDM) features [38], 19 first order statistics features, and 14 shape-based features. All features were determined from the CT image and its wavelet coefficients, but for shape-based features. Each CT image was decomposed to 8 wavelet coefficients. Details of determined features from each texture-based CT radiomic are provided in the Supplementary Data (Table S1).

In total, 851 features were determined for each patient from the CT image (107 features) and its wavelet coefficients (each coefficient 93 features).

### 2.4. Clinical Features

Clinical features include molecular features, subject demographic features, and histopathological features. We considered Estrogen-receptor (ER) and progesterone-receptor (PR) status, HER-2/neu status, age, pre-treatment tumor size, Nottingham grade, and nodal status (TMN staging) as clinical features in this study.

### 2.5. Machine Learning

In this study, we applied the leave-one-out technique to split train and test sets. Values beyond three standard deviations were considered outliers. Z-score standardization was applied to scale the data, ensuring a mean of zero and a standard deviation of one. Feature selection was performed to reduce the dimensionality of the data, decrease the probability of overfitting, reduce computation time, and address the curse of dimensionality. For feature selection, we proposed a hybrid technique that combined two filter and wrapper strategies. A support vector machine (SVM) with a radial basis function (RBF) kernel was employed to classify responders and non-responders. The SMOTE technique was used to address the imbalance in the training data by oversampling the minority group [39].

#### Proposed Hybrid Feature Selection

The performance of a classifier is directly affected by the dimensionality of the data. Feature extraction and feature selection are two common techniques for dimensionality reduction. However, feature selection offers better interpretability than feature extraction. The aim of feature selection is to remove redundant features while retaining all essential ones. Feature selection strategies are categorized into filter, wrapper, and embedded techniques [40]. In the filter strategy, feature selection is independent of the classifier and data. In the wrapper strategy, the top features are selected based on the performance of the classifier. In the embedded strategy, feature selection is integrated into the learning process, as seen in algorithms like decision trees [40]. In this study, we proposed a hybrid feature selection technique that uses a filter strategy to identify all independent features, followed by a wrapper strategy to select the top features. Thus, our approach consists of a two-phase feature selection process. In the first phase (filter strategy), all features are ranked based on the information gain criterion. Then, we test the independence of features by adding them sequentially. In this technique, the first feature is considered as the selected feature, and additional features are added one by one. Independence is checked after each feature is added. A full-column rank matrix indicates that all columns are independent. Based on matrix theory, a matrix can be partitioned into column space and null space, with all essential information embedded in the column space. To this end, Moslemi et al. [41]. combined perturbation theorem with matrix theory for feature selection since perturbation of column space, which carries the most informative features, has much less variation by perturbation than null space, which carries all dependent features. Saberi et al. proposed a feature selection technique based on basis construction [42]. They proposed an iterative algorithm to find those features which span all features. The algorithm works based on the matrix rank theorem, according to which the algorithm would be stopped if the number of selected features reached the rank of matrix. Afshar et al. proposed Singular-Vectors Feature Selection which is based on matrix partitioning and rank theorem [43].

Using singular value decomposition (SVD), we can access the column space and extract the first *r* columns of matrix *U*, where *r* is the rank of the matrix (matrix *U* is obtained via SVD) [44]. However, the main challenge is that we cannot directly identify the features corresponding to the column space. To address this challenge, we proposed a feature selection technique in which features were first sorted based on information gain. Independent features are then sequentially selected by checking the rank of the matrix for each added feature. To clarify this technique, if we have four features F_1, F_2, F_3, and F_4, we ranked a feature based on information gain and we have F_1 > F_2 > F_3 > F_4. We consider F_1 as a selected feature and in the next step we add F_2. Now we have {F_1, F_2} which is a feature matrix with “*n*” samples and 2 features. In this step, we check the rank of this feature matrix and if we have a full rank matrix we keep F_2, otherwise F_2 will be removed. We repeat this procedure for all features to keep all independent features. It should be noted that although F_2 is ranked as a second top feature, maybe F_2 is highly correlated with F_1 and must be removed to minimize redundancy. Based on matrix theory, the number of independent columns of a matrix is the column rank of a matrix. The aim of feature selection is to obtain a subset of features that serves as a good approximation of the entire feature set. For a given data X∈R^(*n* × d)^, X′∈R^(*n* × k)^ is a subset of X such that X (X′⊂X) is spanned by X′(k << d). In our proposed approach, X′ is abstained based on rank of matrix using an iterative-based technique. Finding all independent features, which are sorted based on information gain, provides a good approximation of all features.

We summarized the first phase in Algorithm 1.
**Algorithm 1.** Removing all dependent and redundant features**Input:** Data
$X\in R^{n\times d}$, where
n and
d are samples and features, respectively. 1-Rank the features based on information gain:
$\left\{F_{1},F_{2},\ldots ,F_{d}\right\}$, where
F represents a feature and
$F_{1}>F_{2}>\ldots >F_{d}$ in terms of information gain. 2-Select the first feature as selected feature:
$F_{1}$,
$X^{\prime }=\{F_{1}\}$ 3-**for** 
i=2:d, #For all features
$\left\{F_{2},\ldots ,F_{d}\right\}$ 4-Obtain rank
$X^{\prime }={\{F}_{1},F_{i}\}$ 5-**if** it is full rank 6-
$X^{\prime }\leftarrow X^{\prime }\cup \{F_{i}\}$: keep
$F_{i}$ 7-**end if** 8-**end for** **Output:** Selected features,
$X^{\prime }\in R^{n\times d^{\prime }}$, where
$d^{\prime }$ shows the number of selected features after removing redundant features and
$d^{\prime }<d$.

**Figure 2:**
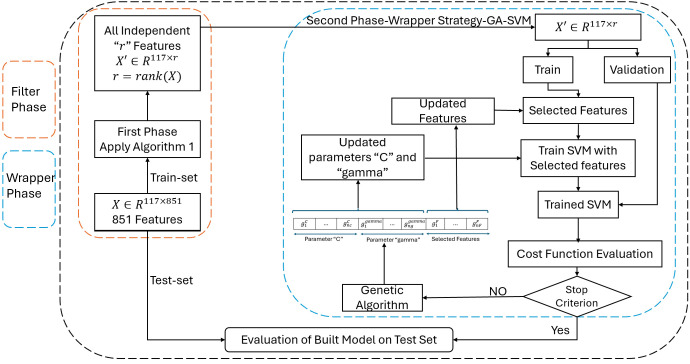
This figure shows the procedure of proposed hybrid feature selection. In the first phase, which is placed in orange box, a filter-based technique is applied to remove all dependent features. In second phase, which is placed in blue box, a GA algorithm is applied to not only find top features but also tune the SVM hyperparameters.

In the second phase (wrapper strategy), the genetic algorithm (GA) is coupled with the SVM to not only obtain top features but also optimize the hyperparameters of the SVM. The SVM has two hyperparameters including “C” and “gamma”; “C” controls the complexity of the algorithm and “gamma” is the radius of the kernel. The superiority of GA is that the optimum number of selected features is obtained as well. In the second phase, a chromosome (solution) of the GA was partitioned into three parts including a parameter “C” part, parameter “gamma”, and selected features. A GA is a discrete algorithm and its chromosome has two values “0” and “1”. For feature selection, parts of chromosome, “1” and “0”, represent selected and unselected features, respectively. For the two hyperparameters “C” and “gamma”, a binary value at end of the iteration is transformed into a decimal value. The GA searches space based on an exploration and exploitation strategy. Exploration is performed by a mutation operator with the aim of achieving a global search, and exploration is performed by a crossover operator with the aim of achieving a local search. The schematic of the proposed feature selection technique is shown in Figure 2.

We defined cost function based on the accuracy of the SVM. The cost function for the GA to select features and optimize the SVM hyperparameters is defined as follows:

(1) μ(1 − Classification Accuracy) + (1 − μ)g

where 0 < μ < 1 is a parameter to keep a balance between classification accuracy and selected features. g = N_f/N_t ≤ 1 is the ratio of selected features, and the goal of this cost function is to minimize the number of selected features and maximize the classifier accuracy consideration. N_f is the number of selected features and N_t is the number of total features, respectively. (1) Objective function minimizes the classification and the number of selected features simultaneously. The GA parameters are as follows: population size (number of chromosomes) = 100, crossover rate = 0.6, and mutation rate = 0.1.

The effectiveness of our proposed feature selection approach lies in its ability to identify the optimal number of features that maximize classification accuracy. In contrast, other feature selection techniques merely rank features and select a fixed number, such as the top 5 or top 10, or rely on greedy search methods, which often result in suboptimal local solutions. Different features are selected in each run; therefore, the histogram of selected features is analyzed, and the most frequently occurring features are chosen.

### 2.6. Different Models Based on Feature Type

In this study, we considered three models including only imaging features, only clinical features, and a combination of imaging and clinical features. We had 851 radiomic CT features and 7 clinical features including HER2, ER, PR, age, pre-treatment tumor size, Nottingham grade, and nodal status (TMN staging).

### 2.7. Statistical Analysis

We applied Chi-square and *t*-test for categorical and continuous features, respectively. In this study, we used MATLAB Statistics and Machine Learning Toolbox™ ((ver. 9.6.0.1072779 R2020b), The MathWorks, Inc., Natick, MA, USA).

### 2.8. Evaluation Metric and Implementation

To evaluate our predictive model, we considered accuracy, F1-score, area under curve (AUC), and balanced-accuracy metrics. Balanced accuracy is the average of sensitivity and specificity. PyRadiomics version 3.0.1 was used to extract radiomics features. The we built a machine learning pipeline including outlier detection, feature normalization, feature selection, and classification using MATLAB R2020b (MathWorks Inc., Natick, MA, USA). The codes were implemented on Intel (R) Core (TM) i7-1065G7 CPU 1.5 GHz CPU and 16 GB Ram.

## 3. Results

Among the 117 women with mean age 52 ± 11 in this study, *n* = 82 (70%) women had either a partial or complete pathological treatment response and we recorded no treatment response based on RECIST [34] criterion for *n* = 35 (30%) women. Histopathology for patients were invasive ductal carcinoma (IDC), invasive lobular carcinoma (ILC), and metaplastic carcinoma (IMC). In this study, the majority of enrolled patients was IDC and the minority was ILC and IMC. In terms of receptor, progesterone (PR+) receptor was the major molecular attribute in enrolled patients, whereas positive Her2/Neu (HER2+) receptor and triple negative tumor (ER−, PR−, HER2−) were realized as minor molecular features in patients with LABC. Additionally, the monoclonal antibody Trastuzumab (Herceptin) (TRA) (with HER2+) tumors was applied for LABC patients. Additionally, positive estrogen (ER+) was major in patients. Doxorubicin (Adriamycin), cyclophosphamide followed by paclitaxel (Taxol) (AC-T), or 5-fluorouracil, epirubicin, cyclophosphamide followed by docetaxel (FEC-D), doxorubicin, cyclophosphamide followed by docetaxel (Taxotere) (AC-D) and paclitaxel and cyclophosphamide (TC) were the utilized chemotherapy regimens for LABC treatment. Furthermore, the tumor size varied on average from 5.2 cm to 1.4 cm and from 5.6 cm to 6 cm for responders and non-responders, respectively. It should be mentioned that we did not have therapy modification based on imaging during this observational study. In Table 1, we listed all the pathological and clinical characteristics of enrolled patients with LABC.

In total, 858 radiomic and clinical features were determined. We had 7 clinical features and 851 radiomics features. For the first model, which means only clinical features were considered, Nottingham Grade, ER, PR, and HER were selected features and the classifier obtained an accuracy = 0.85 (F1-score = 0.89, balanced accuracy = 0.76, and AUC = 0.73). For the second model, only imaging features were considered, first order Kurtosis original image, GLCM cluster shade LLL of the original image, GLRLM grey level variance of the original image, first order robust mean absolute deviation HLL, wavelet-LLH-GLDM-dependence entropy were the selected features and the classifier achieved an accuracy = 0.75 (F1-score = 0.73, balanced accuracy = 0.72, and AUC = 0.70). A combination of clinical features and CT radiomic features formed the last model. ER, PR, HER, wavelet-LLH-GLDM-dependence entropy, GLCM cluster shade LLL of the original image, and GLRLM grey level variance of the original image were the selected features and the classifier achieved an accuracy = 0.88 (F1-score = 0.90, balanced accuracy = 0.80, and AUC = 0.78). Table 2 shows the selected features for each model. Table 3 shows the performance of the SVM classifier for each model.

We applied a two-sided independent *t*-test (with Bonferroni correction for multiple comparisons) to assess group differences in continuous variables, and a Chi-square test for categorical variables. Groups were defined based on response status (responders and non-responders) to NAC. Levene’s test was applied to assess homogeneity of variance, which indicated equal variances (*p* > 0.05), allowing the use of the standard *t*-test. The results showed statistically significant differences in ER and HER2 between the groups, with Bonferroni-adjusted *p*-values of 0.01 and 0.0003, respectively. Therefore, ER and HER2 are significantly associated with response status.

**Figure 3:**
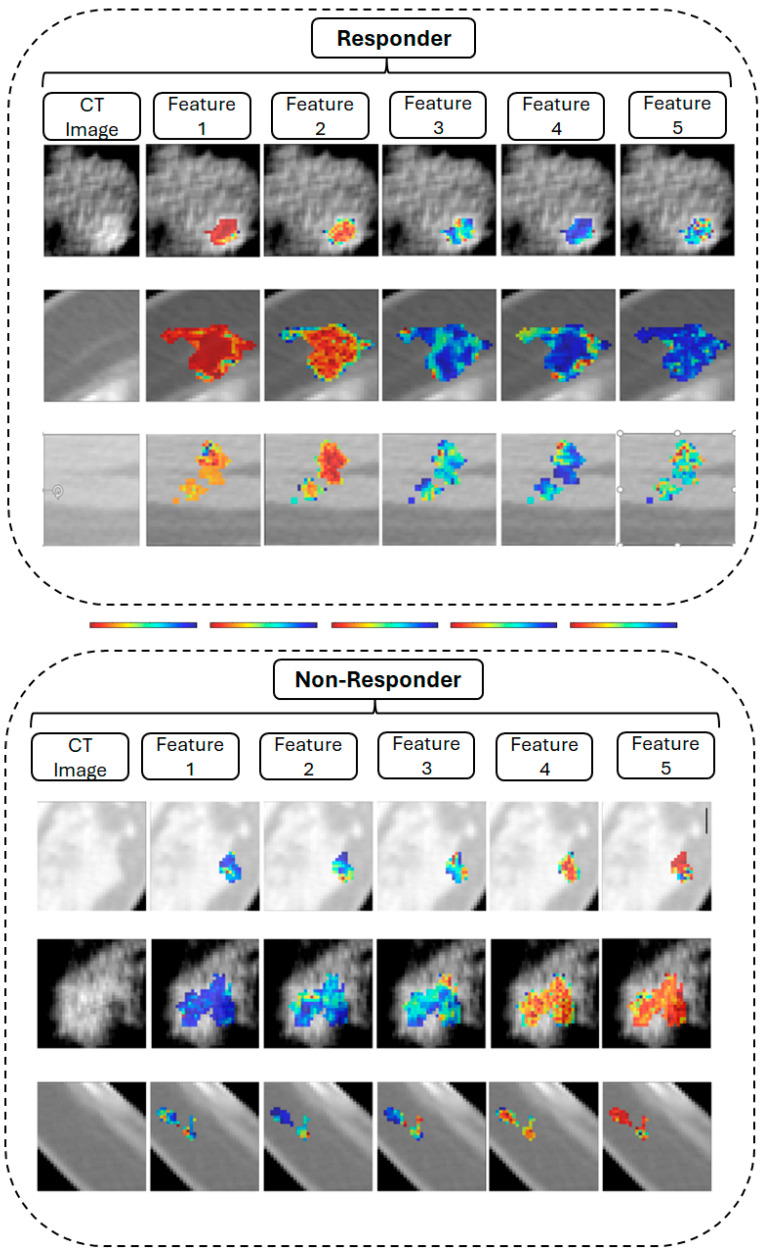
This figure shows the parametric maps for the responder and non-responder groups. The parametric maps show first order Kurtosis original image (Feature 1 with range [0–4]), GLRLM grey level variance of original image (Feature 2 with range [0–35]), first order robust mean absolute deviation HLL (Feature 3 with range [0–18]), wavelet-LLH-GLDM-dependence entropy (Feature 4 with range [0–4]), and GLCM cluster shade LLL (Feature 5 with range [−14,000–0]). The scale bar represents 1 cm.

Additionally, for the selected features in Model 2, we showed CT images with parametric feature maps for both responder and non-responder patients in Figure 3.

We applied the DeLong test, a statistical method used to compare the AUC of two correlated ROC curves [45]. To this end, we compared the AUCs of Model 1 (clinical features), Model 2 (CT radiomic features) and Model 3 (combination of clinical and CT radiomics). For all three models, *p*-values of Delong test were statistically significant (*p*-values < 0.001).

We compared the proposed feature selection technique with LASSO feature selection as a conventional feature selection method. Table 4 shows the performance of the SVM classifier for the proposed feature selection technique and LASSO feature selection when a combination of clinical and CT radiomics features are used. Based on Table 4, the proposed method outperforms the LASSO technique.

## 4. Discussion

In this study, we demonstrated that combining clinical features and CT-based textural features at different frequency levels, along with a robust hybrid feature selection approach, can predict the response to NAC in patients with LABC based on RECIST and histopathological criteria. Although our dataset included only 117 patients, we employed an SVM, a method robust to overfitting, and optimized its hyperparameters using a GA. The proposed hybrid feature selection improved the performance of the machine learning model in predicting NAC response by selecting the most discriminative features. In most studies on treatment response prediction, a fixed number of top features (e.g., *k* = 5, 7, 10) is considered [29,30], or sequential feature selection (SFS) is applied [21]. However, neither technique is optimal, as the optimal number of features is not determined. To evaluate the effectiveness of our proposed technique, we utilized MRMR feature selection combined with SFS (MRMR-SFS), achieving a balanced accuracy of 76%, (Table S2 in the Supplementary Data provides details on MRMR-SFS for all three models). In contrast, our hybrid approach achieved a balanced accuracy of 80%, highlighting its superior performance compared to MRMR-SFS. The selected features for each model using MRMR-SFS are listed in Table S3 in the Supplementary Data.

However, in this study, the proposed feature selection technique can find the exact optimum number of features, and this plays a significant role for machine learning algorithms. Knowing the optimum number of features and those top features leads to the improvement of a learning algorithm. In most of the studies, features are firstly ranked, and then a top k-feature is considered, whereas Nie et al. [46] showed that a k-feature is not necessarily the top k-feature. Loosely speaking, if we have four features with the importance order F_1 > F_2 > F_3 > F_4, the top two features are F_1 and F_2 based on importance, whereas the combination of F_1 and F_4 can provide better discrimination in subspace (projected space). In this regard, the GA algorithm is able to determine the top k-feature which provides the maximum separation between the responder group and non-responder group.

Although suboptimal convergence is the main challenge and drawback of a GA, the first phase of feature selection, in the proposed hybrid feature selection, removes all local optima. In other words, all redundant features play the role of local optima, and they are removed in the first phase using the proposed filter strategy. Therefore, the quality of searching space is increased by the first phase and the GA is able to find optimum solution without concern. A GA is a discrete algorithm and that is why it is an appropriate algorithm for a feature selection task. A continuous metaheuristic algorithm such as particle swarm optimization (PSO) cannot be directly applied for feature selection. Moslemi et al. applied the binary version of PSO for feature selection to identify a patient with chronic obstructive pulmonary disease (COPD) and asthma [47]. In another study, mushroom reproduction optimization as a continuous metaheuristic algorithm applied for gen selection in a genomic dataset using key random trick [48]. Jointly performed supervised feature selection and hyper-parameter tuning improve the performance of machine learning models [49].

Hybrid techniques improve the performance of metaheuristic algorithms. A major drawback of these algorithms is their tendency to suboptimal convergence. In the feature selection problem, correlated and dependent features can act as local optima. Therefore, the first phase of the algorithm filters out all dependent features in order to enrich the search space for metaheuristic algorithms. In our study, we used matrix rank to identify all independent features and then employed a GA to select the most discriminative subset of features, ultimately achieving the best performance with an SVM classifier.

In terms of a hybrid feature selection approach [50], the article proposed a hybrid feature selection based on Reduced Row Echelon Form (RREF) and a GA to predict healthcare utilization of patients with COPD using quantitative CT biomarkers. RREF aims to transform a matrix to a simpler form which leads to the loss of information. A serious point that must be noted is that the performance of the classifier is directly affected by the loss of information. Additionally, RREF is appropriate for a square matrix whereas, in our dataset, the number of features was much greater than the number of samples and we had an undetermined system equation problem (rectangular matrix). Nevertheless, there is no concern about information loss in our proposed method.

Although most of the studies employed the grid search technique to tune the hyperparameters of the classifier [21,27,29,32,33], suboptimal convergence is the main challenge of the grid search technique. In the proposed technique, the hyperparameters of the SVM are tuned simultaneously with features selection using a GA in wrapper phase. Since a GA follows the exploration and exploitation policies to find the optimum solution, there is no concern about local optimum for hyperparameters of the SVM.

In this study, we had 851 radiomic features, which were determined from the CT image and its wavelet coefficients, and seven clinical features. Results showed that the combination of clinical and radiomic features provides the better performance. The optimum number of features was six and features were ER, PR, HER, wavelet-LLH-GLDM- dependence entropy, GLCM cluster shade LLL of original image, and GLRLM grey level variance of original image. ER, PR, and HER were clinical features, and wavelet-LLH-GLDM-dependence entropy, GLCM cluster shade LLL of original image, and GLRLM grey level variance of original image were radiomic imaging features. Each of the radiomic features refers to tumor heterogeneity which directly impacts the treatment outcome. For example, GLRLM grey level variance of the original image shows more diverse grey levels in texture, indicating biological complexity in the tumor.

Studies have shown a significant correlation between cellular microstructure characteristics and response to treatment [31,51,52,53]. Although there is a correlation between the voxel intensity of CT images and changes in cellular microstructure due to variations in the attenuation coefficient of X-rays [50], the details of cellular structures cannot be visualized or extracted using clinical CT due to its resolution limitations. Radiomic features offer insights into the microstructure of tumor tissue that cannot be obtained through CT alone, primarily due to these resolution constraints.

CT image voxels can be quantified using radiomic features to capture spatial variations that represent tumor structure information and can be directly utilized to predict response to treatment before initiation. Therefore, radiomic features can be employed to train a machine learning model to distinguish between LABC patients who responded to NAC treatment and those who did not. We proposed a machine learning pipeline to predict treatment response in patients with LABC. This pipeline includes outlier removal, feature normalization, feature selection, classifier hyperparameter tuning, and oversampling of the minority class.

A classifier is significantly affected by imbalanced data, which biases the learning algorithm toward the majority class, making the prediction of the minority class challenging. To address this, we applied the SMOTE technique to oversample the minority class and improve the model’s learning during the training phase. Balanced accuracy was used to evaluate the predictive model’s effectiveness in predicting the minority class. As shown in Table 3, the proposed method is a robust technique for minority class prediction. The effectiveness of clinical features in predicting treatment response has been investigated [54,55]. Li et al. [54] demonstrated that negative ER/PgR, negative Topo-II, and positive nm23-H1 are statistically significant predictors of treatment response. In our study, ER, PR, and HER clinical features were identified as top features in Model 3 (a combination of clinical features and CT radiomic features).

In terms of the ability of radiomic features to predict response to NAC, Sadeghi-Naini et al. [56] utilized radiomic features, including GLCM derived from diffuse optical spectroscopic (DOS) images, to distinguish responders from non-responders to NAC in patients with LABC. Their findings indicated that NAC response could be predicted at an early stage using DOI-based radiomic features and mean-value parameters. In another study, radiomic features of quantitative ultrasound (QUS) and image quality features were used to differentiate responders from non-responders in patients with LABC [31]. Tren et al. [57] conducted a study applying logistic regression, naive Bayes, and KNN classifiers to classify responders and non-responders in patients with LABC using radiomic features derived from DOS.

Dastjerdi et al. [32] conducted a study to demonstrate the effectiveness of GLCM features derived from CT in predicting treatment response for 72 patients with LABC. They ranked features using MRMR and employed various classifiers, including SVM, decision trees (DT), multilayer perceptron (MLP), and random forest, to classify responders and non-responders. In another study, second-order radiomic features (GLCM) from CT were used to predict response to NAC in patients with LABC [33]. Second-order GLCM features involve developing a new feature map based on first-order GLCM, from which quantitative features are derived.

Teruel et al. [26] investigated the prediction of NAC response for 58 patients with LABC using 16 GLCM-based textural radiomic features from dynamic contrast-enhanced MRI (DCE-MRI). Cheng et al. [58] employed radiomic features from 18F-FDG PET/CT to differentiate responders from non-responders to NAC in 61 patients with LABC. The features included maximum standardized uptake value, metabolic tumor volume, total lesion glycolysis, entropy, coarseness, and skewness.

When comparing our study with the above studies, our dataset was larger, enabling better generalization of the machine learning model. Additionally, while previous studies considered only imaging-based radiomic features, we combined clinical and radiomic features. Table 3 demonstrates the efficacy of integrating clinical features with CT radiomic features, resulting in a combined model with a balanced accuracy 8% higher than that of the CT radiomic model alone. Furthermore, the above studies only utilized GLCM features from the original images, whereas we derived GLSZM, GLRLM, NGTDM, and GLDM features in addition to GLCM from the original CT image and its wavelet coefficients.

In this study, we extracted 3D radiomic features to capture 3D information. The geometric information of the tumor can only be obtained using 3D textural features. Unlike 2D radiomic features, which fail to preserve the tumor’s geometrical information, 3D radiomic features analyze tumor dependencies across slices. The effectiveness of 3D textural features has been reported [59]. In our 3D model, we defined a 3D region of interest (ROI), allowing the tumor’s topology to provide critical information for analyzing treatment response.

Recently, deep learning has gained considerable attention in treatment response prediction. Falou et al. [60] applied a deep learning-based approach using pretrained ResNet and QUS to predict response to NAC in patients with LABC. However, there are concerns regarding the use of deep learning. First, deep learning models lack interpretability, whereas our study explicitly identified the selected features and provided their clinical interpretation. Second, pretrained models, such as those trained on the ImageNet dataset, are based on general images, which lack correlation with medical images. Third, pretrained models require three input channels, while CT images typically consist of over 100 slices. Selecting only three slices leads to significant information loss. Lastly, deep convolutional neural networks (CNNs) lack an attention mechanism and cannot capture long-range dependencies or relationships in data.

The aim of this study was to design a machine learning pipeline to predict NAC response for patients with LABC before treatment initiation using CT imaging. Oncologists can utilize this model to predict treatment response in advance, allowing for treatment modification and optimization to maximize effectiveness. This artificial intelligence-based model aids oncologists in improving treatment outcomes.

### Limitation

This study has a few limitations. First, the dataset size was small, and a larger dataset would improve generalization. Second, no contrast agent was used during CT imaging, which could have enhanced image quality. The segmentation of the tumor was done by a single radiologist specializing in breast radiology. Lastly, all patients were from a single center contributing to consistent data and improved machine learning performance. However, this practice may reduce the generalizability of the model and its results.

## 5. Conclusions

In this study, we proposed a machine learning model with hybrid feature selection to predict NAC response for patients with LABC using CT radiomic and clinical features. Radiomic features were derived from CT images and their wavelet coefficients. A two-step feature selection process was employed: in the first step, an algorithm was developed to identify all independent features based on the rank of matrix theorem. In the second step, a GA was used to select the most informative features and optimize the hyperparameters of the SVM classifier. The results indicate that combining clinical features with CT radiomic features is an effective approach to enhance the performance of machine learning models in predicting NAC response for patients with LABC.

## Figures and Tables

**Table 1:** Summary of pathological and clinical characteristics of 117 patients.

Characteristics	Responders: 82 (70%) Mean (Std)	Non-responders: 35 (30%) Mean (Std)
Age	52 (11)	54 (10)
**Initial tumor size**	5.2 (2.5) cm	5.6 (2.7) cm
**Residual tumor size**	1.4 (2.4) cm	6 (5.5) cm
**Histology**	Percentage (count)	
IDC	58 (70)	23 (65)
ILC	1 (1)	4 (11)
IMC	3 (3)	2 (5)
**Molecular features**	Percentage (count)	
ER+	42 (51)	29 (82)
PR+	37 (45)	24 (68)
HER2+	28 (34)	9 (26)
ER−/PR−/HER2−	22 (27)	4 (11)
ER+/PR+/HER2+	15 (18)	6 (17)
ER+/PR+/HER2−	22 (27)	20 (57)
ER−/PR−/HER2+	15 (18)	4 (11)

Std = standard deviation, IDC = invasive ductal carcinoma, ILC = invasive lobular carcinoma, IMC = invasive metaplastic carcinoma, ER = estrogen, PR = progesterone.

**Table 2:** The selected features for each model. This Table shows the selected features by proposed technique for three models including Model 1-clinical features, Model 2-radiomic features, and Model 3-combination of clinical and radiomic features.

Model 1: Clinical Features	Model 2: Radiomic Features	Model 3: Combination of Clinical and Radiomic Features
Nottingham Grade	First order Kurtosis original image	ER
ER	GLRLM grey level variance of original image	PR
PR	First order robust mean absolute deviation HLL	HER
HER	Wavelet-LLH-GLDM- Dependence Entropy	wavelet-LLH-GLDM- dependence entropy
	GLCM cluster shade LLL of original image	GLCM cluster shade LLL of original image
		GLRLM grey level variance of original image

**Table 3:** Performance of the outcome prediction for all three models using leave-one-out data splitting and SVM classifier with RBF kernel.

	Accuracy (%)	F1-Score (%)	AUC (%)	Balance Accuracy (%)
**Model 1:**				
Clinical Features	85	89	73	76
**Model 2:**				
CT Radiomic Features	75	73	70	72
**Model 3:**				
Combination of Clinical and CT Radiomics	88	90	78	80

**Table 4:** Performance of outcome prediction using the proposed method and LASSO with an SVM classifier and RBF kernel. Grid search was applied to tune the SVM hyperparameters for the LASSO technique.

	Accuracy (%)	F1-Score (%)	AUC (%)	Balance Accuracy (%)
**Proposed Technique**	88	90	78	80
**LASSO**	86	87	75	77

## Data Availability

Data are available upon request (contact the Czarnota Lab at Sunnybrook Health Sciences Centre).

## Supplementary Materials

The following supporting information can be downloaded at: https://www.mdpi.com/article/10.3390/cancers17172738/s1, Table S1. Extracted radiomic features of original image and wavelet coefficients. Table S2. Performance of the outcome prediction for all three models using MRMR-SFS feature selection and SVM classifier with RBF kernel. SVM hyperparameters were tuned by grid search technique. Table S3. The selected features for each model. This table shows the selected features by MRMR-SFS technique for three models including Model 1-clinical features, Model 2-Radiomic features, and Model 3-combination of clinical and radiomic features.
