# Supplementary material for: Hybrid Feature Selection for Predicting Chemotherapy Response in Locally Advanced Breast Cancer Using Clinical and CT Radiomics Features: Integration of Matrix Rank and Genetic Algorithm
Source: Cancers (Basel). 2025 Aug 23;17(17):2738. doi: 10.3390/cancers17172738 (PMC12427367; doi:10.3390/cancers17172738)
Supplement: Supplementary file 1 [file cancers-17-02738-s001.zip › cancers-3750486-supplementary.pdf]

Table S1. Extracted radiomics features of original image and wavelet coefficients.

Radiomics Type	Features	Radiomics Features
First Order Features:	Energy<br>Total Energy<br>Entropy<br>Minimum<br>10th percentile<br>90th percentile<br>Maximum<br>Mean<br>Median	Interquartile Range<br>Range<br>Mean Absolute Deviation (MAD)<br>Robust Mean Absolute Deviation<br>Root Mean Squared (RMS)<br>Skewness<br>Kurtosis<br>Variance<br>Uniformity
Shape Features:	Elongation<br>Flatness<br>Least Axis Length<br>Major Axis Length<br>Maximum 2D Diameter Column<br>Maximum 2D Diameter Row<br>Maximum 2D Diameter Slice	Maximum 3D Diameter<br>Mesh Volume<br>Minor Axis Length<br>Sphericity<br>Surface Area<br>Surface Volume Ratio<br>Voxel Volume
GLCM:	Autocorrelation<br>Joint Average<br>Cluster Prominence<br>Cluster Shade<br>Cluster Tendency<br>Contrast<br>Correlation<br>Difference Average<br>Difference Entropy<br>Difference Variance<br>Dissimilarity<br>Joint Energy	Joint Entropy<br>Homogeneity 1<br>Homogeneity 2<br>Informational Measure of Correlation (IMC) 1<br>Informational Measure of Correlation (IMC) 2<br>Inverse Difference Moment (IDM)<br>Maximal Correlation Coefficient (MCC)<br>Inverse Difference Moment Normalized (IDMN)<br>Inverse Difference (ID)<br>Inverse Difference Normalized (IDN)<br>Inverse Variance<br>Maximum Probability<br>Sum Average<br>Sum Variance<br>Sum Entropy<br>Sum of Squares
GLRLM:	Short Run Emphasis (SRE)<br>Long Run Emphasis (LRE)<br>Gray Level Non-Uniformity (GLN)<br>Gray Level Non-Uniformity Normalized (GLNN)	Run Percentage (RP)<br>Gray Level Variance (GLV)<br>Run Variance (RV)<br>Run Entropy (RE)<br>Low Gray Level Run Emphasis (LGLRE)

	Run Length Non-Uniformity (RLN)<br>Run Length Non-Uniformity Normalized (RLNN)<br>Long Run Low Gray Level Emphasis (LRLGLE)<br>Long Run High Gray Level Emphasis (LRHGLE)	High Gray Level Run Emphasis (HGLRE)<br>Short Run Low Gray Level Emphasis (SRLGLE)<br>Short Run High Gray Level Emphasis (SRHGLE)<br>Long Run Low Gray Level Emphasis (LRLGLE)<br>Long Run High Gray Level Emphasis (LRHGLE)
GLSZM:	Small Area Emphasis (SAE)<br>Large Area Emphasis (LAE)<br>Gray Level Non-Uniformity (GLN)<br>Gray Level Non-Uniformity Normalized (GLNN)<br>Size-Zone Non-Uniformity (SZN)<br>Size-Zone Non-Uniformity Normalized (SZNN)<br>Zone Percentage (ZP)<br>Gray Level Variance (GLV)	Zone Variance (ZV)<br>Zone Entropy (ZE)<br>Low Gray Level Zone Emphasis (LGLZE)<br>High Gray Level Zone Emphasis (HGLZE)<br>Small Area Low Gray Level Emphasis (SALGLE)<br>Small Area High Gray Level Emphasis (SAHGLE)<br>Large Area Low Gray Level Emphasis (LALGLE)<br>Large Area High Gray Level Emphasis (LAHGLE)
GLDM:	Small Dependence Emphasis (SDE)<br>Large Dependence Emphasis (LDE)<br>Gray Level Non-Uniformity (GLN)<br>Gray Level Non-Uniformity Normalized (GLNN)<br>Dependence Non-Uniformity (DN)<br>Dependence Non-Uniformity Normalized (DNN)<br>Gray Level Variance (GLV)	Dependence Variance (DV)<br>Dependence Entropy (DE)<br>Dependence Percentage<br>Low Gray Level Emphasis (LGLE)<br>High Gray Level Emphasis (HGLE)<br>Small Dependence Low Gray Level Emphasis (SDLGLE)<br>Small Dependence High Gray Level Emphasis (SDHGLE)
NGLDM:	Coarseness<br>Contrast<br>Busyness<br>Complexity<br>Strength	

Table S2. Performance of the outcome prediction for all three models using MRMR-SFS feature selection and SVM classifier with RBF kernel. SVM hyperparameters were tuned by grid search technique.

	Accuracy (%)	F1-score (%)	AUC (%)	Balance Accuracy (%)
**Model 1:**				
Clinical Features	81	85	70	73
**Model 2:**				
CT Radiomics Features	72	68	69	69
**Model 3:**				
Combination of Clinical and CT Radiomics	84	87	73	76

Table S3. The selected features for each model. This Table shows the selected features by MRMR-SFS technique for three models including Model 1-clinical features, Model 2-Radiomics Features and Model 3- Combination of Clinical and Radiomics Features.

**Model 1:** Clinical Features	**Model 2:** Radiomics Features	**Model 3:** Combination of Clinical and Radiomics Features
Nottingham Grade	First order Kurtosis original image	ER
ER	GLCM Cluster Tendency	PR
PR	First order robust mean absolute deviation HLL	HER
HER	NGLDM Gray Level Variance	wavelet-LLH-GLDM-dependence entropy
Nottingham Grade	GLCM cluster shade LLL	GLCM cluster shade LLL of original image
		NGLDM Gray Level Variance
	GLSZM- Size-Zone Non-Uniformity Normalized LLH	Nottingham Grade
